# Hydrated Ionic Liquids Boost the Trace Detection Capacity of Proteins on TiO_2_ Support

**DOI:** 10.1021/acs.langmuir.1c00525

**Published:** 2021-04-16

**Authors:** Yihui Dong, Aatto Laaksonen, Feng Huo, Qingwei Gao, Xiaoyan Ji

**Affiliations:** † Beijing Key Laboratory of Ionic Liquids Clean Process, CAS Key Laboratory of Green Process and Engineering, State Key Laboratory of Multiphase Complex Systems, Institute of Process Engineering, Chinese Academy of Sciences, Beijing 100190, P. R. China; ‡ Energy Engineering, Division of Energy Science, Luleå University of Technology, 97187 Luleå, Sweden; § Department of Materials and Environmental Chemistry, Arrhenius Laboratory, Stockholm University, Stockholm SE-10691, Sweden; ∥ State Key Laboratory of Materials-Oriented Chemical Engineering, Nanjing Tech University, Nanjing 210009, P. R. China; ⊥ Centre of Advanced Research in Bionanoconjugates and Biopolymers, Petru Poni Institute of Macromolecular Chemistry, Iasi 700487, Romania; # State Key Laboratory of Chemical Engineering and School of Chemical Engineering, East China University of Science and Technology, Shanghai 200237, P. R. China

## Abstract

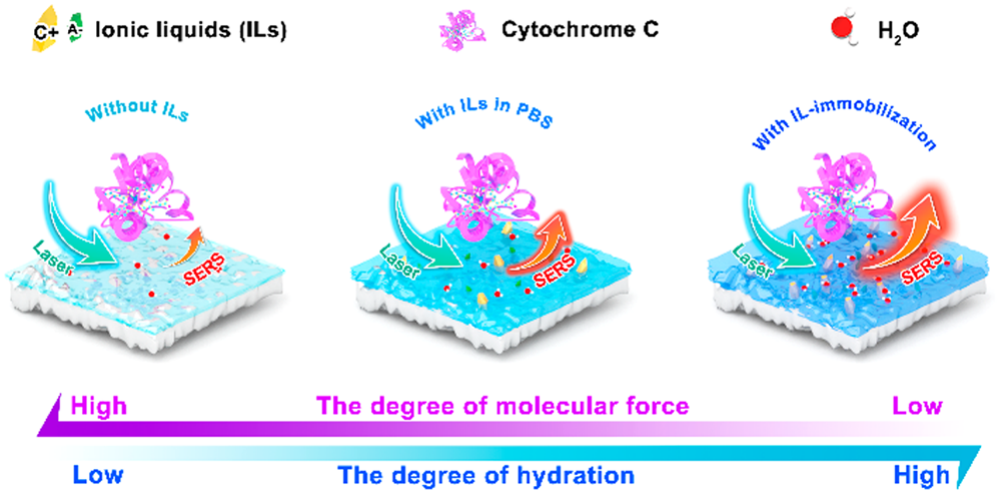

Trace detection based on surface-enhanced Raman scattering (SERS) has attracted considerable attention, and exploiting efficient strategies to stretch the limit of detection and understanding the mechanisms on molecular level are of utmost importance. In this work, we use ionic liquids (ILs) as trace additives in a protein-TiO_2_ system, allowing us to obtain an exceptionally low limit of detection down to 10^–9^ M. The enhancement factors (EFs) were determined to 2.30 × 10^4^, 6.17 × 10^4^, and 1.19 × 10^5^, for the three systems: one without ILs, one with ILs in solutions, and one with ILs immobilized on the TiO_2_ substrate, respectively, corresponding to the molecular forces of 1.65, 1.32, and 1.16 nN quantified by the atomic force microscopy. The dissociation and following hydration of ILs, occurring in the SERS system, weakened the molecular forces but instead improved the electron transfer ability of ILs, which is the major contribution for the observed excellent detection. The weaker diffusion of the hydrated IL ions immobilized on the TiO_2_ substrate did provide a considerably greater EF value, compared to the ILs in the solution. This work clearly demonstrates the importance of the hydration of ions, causing an improved electron transfer ability of ILs and leading to an exceptional SERS performance in the field of trace detection. Our results should stimulate further development to use ILs in SERS and related applications in bioanalysis, medical diagnosis, and environmental science.

## 1 Introduction

Trace detection of proteins plays a major role in a variety of medicinal sectors, and the development of sensitive and specific methods is essential for ultrasensitive bioanalysis and comprehensively understanding the biological processes.^1−4^ The development of highly sensitive enhanced Raman spectroscopic techniques in the last two decades has been remarkable. Surface-enhanced Raman scattering (SERS) has received increasingly attention in the detection of trace amounts of biomolecules due to its extremely high sensitivity and broad tailoring capacity to detect the specific analytes through unique vibrational fingerprints.^5−7^ Compared with other optical detection techniques,^8−10^ SERS shows a great advantage in noninvasive detection properties due to its simple operation and sample preparation. To detect trace biomolecules with SERS, many techniques have been applied to further improve the enhancement performance in a variety of applications.^11,12^

For the trace detection using SERS, an extremely low limit of detection is highly important,^13,14^ which is also a widely used standard for evaluating the SERS performance. Regulating active substrates is often used to enhance the sensitivity (i.e., intensity) and selectivity for trace detection,^1^ especially for the semiconductor-based SERS techniques^15,16^ with a low SERS intensity. Methods such as metal doping, use of composites,^17,18^ as well as structure optimization^19^ and modification^20,21^ have been used to increase the SERS intensity. Intrinsically, the SERS intensity is strongly related to the interactions among the adsorbed molecules and their electron-transfer ability, implying that regulating them is essential to improve the enhancement and to lower the limit of detection. To enhance the sensitivity in SERS-based bioanalysis and biodetection, not only the choice of the substrates, but also the environmental conditions, such as pH,^22,23^ temperature,^24,25^ and ionic strength,^26,27^ can be used to effectively modify the interaction strength of the adsorbed proteins,^28^ leading to an important enhancement of sensitivity.^29^ However, only a handful investigations have been conducted on how to adjust the environmental effects, i.e., pH, temperature, ionic strength, to increase the electron transfer ability. To the best of our knowledge, only limited systematic investigations have been conducted to effectively change the environment for controlling the molecular interactions and electron transfer ability and then achieving a desirable SERS intensity and reaching a low limit of detection.

Using additives has been proposed as a desirable method to adjust the environment and to regulate the interactions between the adsorbed molecules and substrates to modify their properties. Due to their unique physicochemical properties (high ionic conductivity, unique and tunable chemical structures, thermal and chemical stability, etc.), ionic liquids (ILs) have rapidly established themselves in the field of bioanalytical protein chemistry during the last two decades.^30−33^ Using these new green solvents to perform protein detection has received much attention, in particular for identifying the low-abundance analytes in biological samples.^34,35^ Previous works have mainly focused on using ILs as surface modifiers (i.e., ILs-functionalized or modified substrates) to improve the sensitivity and the limit of detection,^36,37^ in which the modification process is usually complex.^38^ To the best of our knowledge, the effect of adding ILs into the SERS measurement as additives to adjust the environment has not been investigated.

With ILs or other additives in the system, the interactions of biomolecules with the substrates and the electron transfer properties may be altered. It is still unclear which one of these two is the primary impactor affecting the SERS performance. Recognizing the interactions between biomolecules and the substrates helps to identify the primary impactor. However, it is inadequate to determine the molecular interactions based on the macroscopic measurements, such as adsorption capacity, retention behavior, electrical signals, etc. It implies that the measurements at the microscale are critically needed. In our previous work, based on the atomic force microscopy (AFM), a method was proposed to determine the molecular force between the protein molecules and different TiO_2_ surfaces.^28,39^ However, applying this method to capture the interactions and to understand the SERS mechanism at the molecular level has not yet been carried out. Meanwhile, the enhancement factor (EF) is an essential parameter and key index in the field of SERS. An accurate calculation of EF is always required, but it is also a long-standing problem. One of the challenges in acquiring EF is how to quantify the amounts of molecules adsorbed on the substrate and excited effectively by the lasers.^40,41^ To address this problem, in our previous work, an AFM-based approach was also proposed to determine the trace amounts of protein molecules on the substrates, thereby making it possible to determine the EF values accurately.^42^

In this work, three systems are created to study the performance of introducing ILs in the SERS systems and clarify the mechanisms. These are the system without ILs for reference, the system with ILs in phosphate-buffered saline (PBS), and the system with ILs immobilized on the substrate. On the basis of the observations that the hydrophilic ILs are better for protein detection than the hydrophobic ILs,^35^ the IL used in this work is choline proline ([Cho][Pro]), which is also inexpensive, biodegradable, and biocompatible and has been commonly employed as a bio-IL.^43^ Due to their intrinsically uniform geometric structure, high stability, and biocompatibility, TiO_2_ nanotube arrays are used as SERS-active substrates in this work.^44,45^ Cytochrome *c* is used as a model probe molecule for SERS measurements due to its electron transfer property as well as its stable charge distribution.^46^ Finally, AFM is used as a powerful tool to detect both adhesion and friction forces for obtaining and verifying the interaction strength to clarify the mechanism at the molecular level.

## 2 Experimental Section

### 2.1 Materials

16-Mercaptohexadecanoic acid (HS(CH_2_)_15_COOH) was provided by Sigma-Aldrich trading Co., Ltd. (Shanghai, China). Trifluoroacetic anhydride (C_4_F_6_O_3_, 98%), *N*,*N*-dimethylformamide (*N*,*N*-DMF, anhydrous), Triethylamine (C_6_H_15_N, 99%), were purchased from J&K Scientific Ltd. (Shanghai, China). Cytochrome *c* (Cyt *c*, *M*_w_: 12.4 kDa, size: 2.6 × 3.2 × 3.3 nm^3^) was purchased from Bio Dee Bio-Tech Co. Ltd. (Beijing, China). The IL Choline proline ([Cho][Pro]) was synthesized according to the process described in the literature.^47^ The substrate of TiO_2_ nanotube array was obtained through the electrochemical anodization of Ti foils (length × width is 2 × 1 cm^2^) at an anodization potential of 35 V following our previous work.^42^ Deionized water was used in all of the experiments.

### 2.2 Preparation of IL-Immobilized TiO_2_

0.01 g ILs ([Cho][Pro]) were dissolved in 60 mL methanol, and then the TiO_2_ substrates were added under stirring for 12 h. The IL-immobilized TiO_2_ samples were placed in a rotary evaporator in a water bath at 60 °C under vacuum to remove methanol and then put into a vacuum drying box at 60 °C for 24 h to ensure the methanol was removed thoroughly. The IL-immobilized TiO_2_ substrate was obtained.

### 2.3 Characterization

The morphology and surface roughness of the samples were characterized by field-emission scanning electron microscopy (FESEM, Hitachi S-4800) and Atomic Force Microscopy (AFM, Bruker ICON). The thermogravimetric analysis (TGA, Model SDT 2960) was used to detect the weight loss of the samples. Fourier Transform infrared spectroscopy (FT-IR) spectra were recorded using an FT-IR spectrophotometer (NEXUS 670). The contact angle meter (DSA 100S, KRUSS GmbH) was used to measure the contact angle between the IL and substrate.

### 2.4 SERS Measurements

The TiO_2_ substrates were soaked in the Cyt *c* solution (5 × 10^–4^ M, pH = 7.2, 0.01 M PBS solution) separately without IL and with ILs adding into the solution (0.01 g), respectively, at 4 °C for 2 h. An inVia Raman microscopy (Renishaw, U.K.) with a 532 nm air-cooled Ar^+^ laser line and a controlled 5 mW laser power was used to obtain the SERS spectrum. Twenty seconds of exposure time, together with two accumulations, was set as the typical spectral collection conditions. The detected SERS signals were fitted and analyzed by the Lorentzian function to obtain the Raman shifts and the intensity using the WiRE software.

### 2.5 AFM Measurements

AFM (Dimension ICON, Bruker) was used to measure the adhesion force at room temperature in the contact mode. The normal spring constant of all the tips was calibrated at the first step, and the normal load signals were transformed from volts (V) into force (N) using the deflection sensitivity of the supported cantilever. The force–distance curve of the ILs immobilized on the TiO_2_ surface was conducted using a bare tip and can be acquired as the force jump during retraction, where the adhesion force represents the pull-off force, which is required to separate the tip after contact.

The protein molecules of Cyt *c* were immobilized on the AFM tips coated with gold (NPG-10, Si_3_N_4_, tip radius: 20 nm) by a chemical attachment with almost the same procedure used for other proteins.^39^ There is a little difference during the last step, where the tips were immersed into 5 mg·mL^–1^ Cyt *c* solution (two tips: one was for the measured-system without ILs, and the other was for the measured-system with the ILs immobilized on TiO_2_ substrate) and with 0.01 g ILs adding into 5 mg·mL^–1^ Cyt *c* solution (one tip for the measured-system with ILs in PBS), respectively. The tips were washed with the PBS solutions and then dried with N_2_. The adhesion forces were obtained according to the force–distance curve approach at the maximal adhesion force upon retraction. About 100 force–distance curves were recorded for analysis at different chosen spots.

The friction force measurements were performed under different load forces using AFM in contact mode with the scan angle at 90° of the tips to the cantilevers long axis for obtaining the lateral force images. The forces were derived from the trace and retrace tracks of lateral force images (2 × 2 μm^2^) and given as an output voltage (V). Then the signals in voltage were transformed into the friction forces (N) according to the torsion of cantilever.^48^ The friction coefficient (μ) was defined and calculated as the proportionality constant of the friction force to the load force.

## 3 Results and Discussion

**Figure 1:**
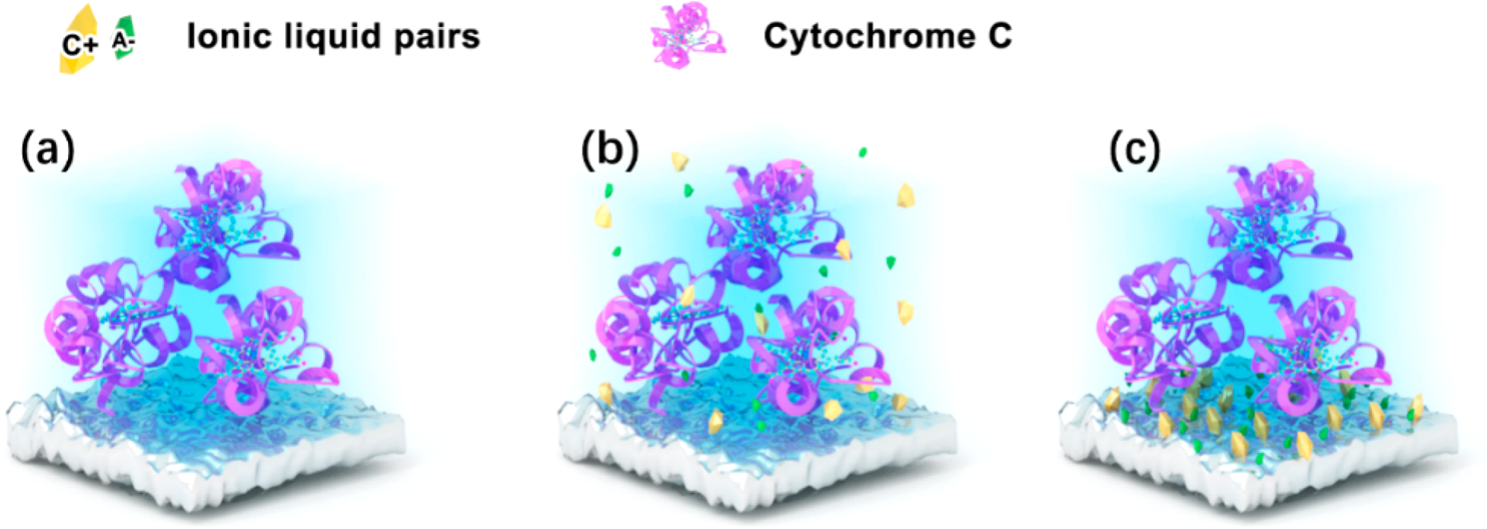
Illustration of three different systems studied in this work: (a) Cyt *c*-TiO_2_ without ILs; (b) Cyt *c*-TiO_2_ with ILs in PBS; and (c) Cyt *c*-TiO_2_ with ILs immobilized on TiO_2_.

In this work, four parts were organized to conduct systematic studies. In the first part, the characterizations of the IL [Cho][Pro], the substrates, and the thickness of IL on the substrates were carried out. The SERS performance of Cyt *c* on TiO_2_ in three different systems was provided together with the report of the limit of detection to highlight the advantages of introducing ILs on the SERS performance in the second part. In the third part, to determine the EF of the SERS performance, the molecular force of Cyt *c* with TiO_2_ was quantified through the combination of the adsorption capacity and adhesion force according to the method developed in our previous work.^42^ The mechanism of the enhancement and molecular force was clarified and discussed, and the friction measurement was used to further verify the mechanism. The three different systems studied in this work are shown in Figure 1 (without ILs, with ILs in PBS, and with ILs immobilized on the TiO_2_ surface, respectively).

### 3.1 Characterization

**Figure 2:**
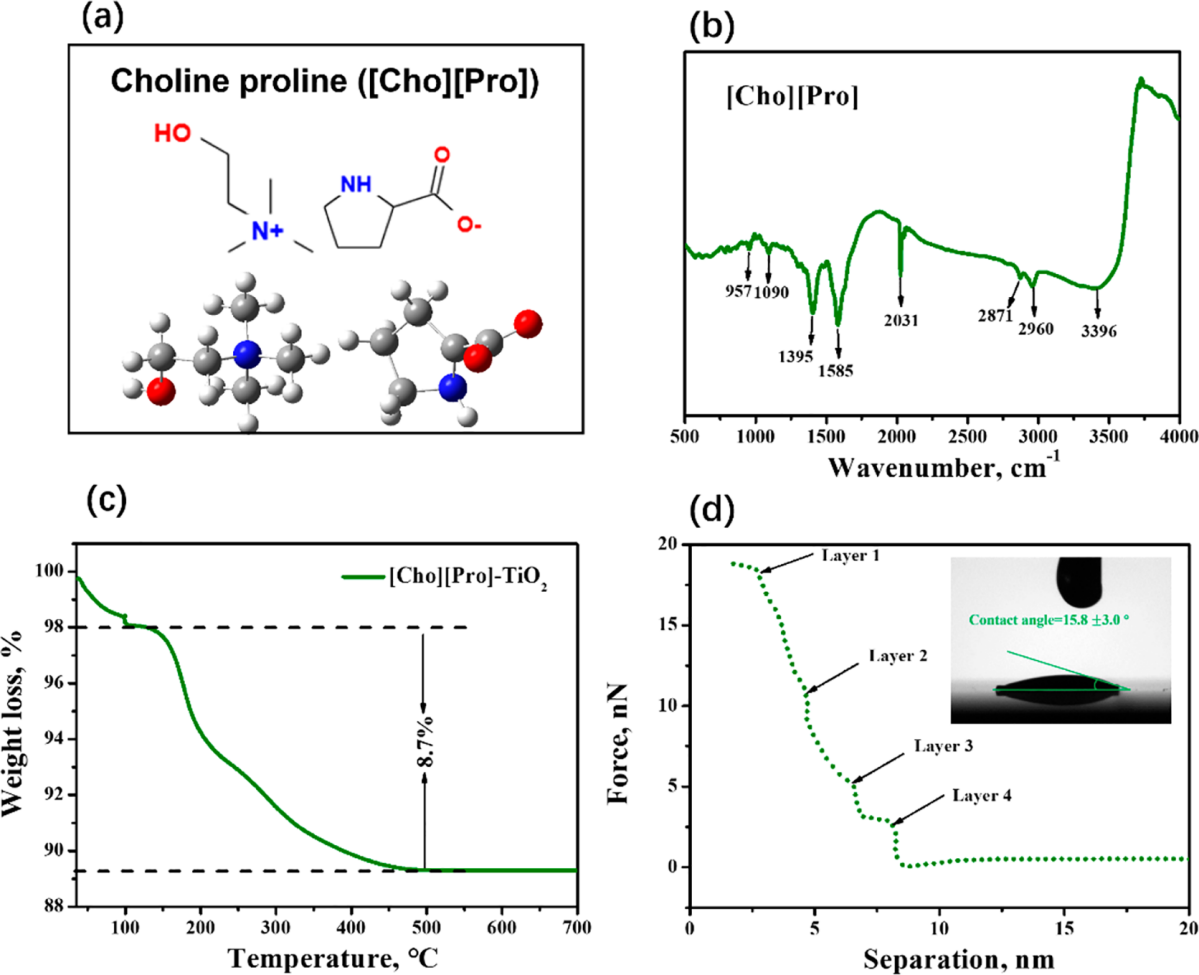
(a) Molecular structure of [Cho][Pro]; (b) FT-IR spectrum of [Cho][Pro]; (c) TGA experiment of [Cho][Pro]-immobilized TiO_2_ sample; (d) force–separation curves of the AFM bare tip with IL-immobilized TiO_2_ nanotube array; inset: contact angle between [Cho][Pro] and TiO_2_ nanotube array.

Figure 2 shows the molecular structure (Figure 2a) and characterization of the IL ([Cho][Pro]). According to the FT-IR spectrum in Figure 2b, the peak at 3396 cm^–1^ represents the stretching vibrations of O–H and N–H, and the peaks at 1585 and 1395 cm^–1^ represent the stretching and torsional vibration of the COO- group, respectively. The peaks at 2871 and 2960 cm^–1^ represent the symmetrical and asymmetrical stretching vibrations of C–H, respectively. The TGA experiments were used to determine the content of [Cho][Pro] immobilized on the TiO_2_ surface, as shown in Figure 2c. The weight loss at the first step in 100 °C represents the free-water loss on the TiO_2_ surface, and the weight loss at the second step ranging from 100 to 500 °C represents the loss of [Cho][Pro] with the reduction of 8.7% approximately. Then the thickness of IL on the TiO_2_ surface was calculated with an approximate value of 8.6 nm, according to the TGA measurements with the consideration of the effective surface area of TiO_2_ after immobilization and the density of IL.^49^

The active substrates used in this work are the TiO_2_ nanotubes, prepared under the anodization voltage with 35 V, where the pore diameter and surface roughness are approximate 65.0 and 42.4 nm, respectively (see Figure S1 of the Supporting Information, SI).

Due to their hydrophilic character of both TiO_2_ and [Cho][Pro], the contact angle between [Cho][Pro] and TiO_2_ is about 15.8° (see Figure 2d inset), indicating the IL could spread out evenly on the TiO_2_ nanotube. Furthermore, the AFM-measured force–distance curve was used to verify the thickness and the layer structure of the ILs immobilized on the TiO_2_ surface. The force–distance curve was converted to the force–separation curve to facilitate the direct estimation of the thickness and inspection of the rigidity of the layered structure.^50^ As shown in Figure 2d, the thickness is about 7.5 nm, which matches well with the values calculated by TGA described in the above text. Meanwhile, the layer thickness is about 1.5 nm, also matching with the size of the [Cho][Pro] molecule.

### 3.2 SERS Measurements

**Figure 3:**
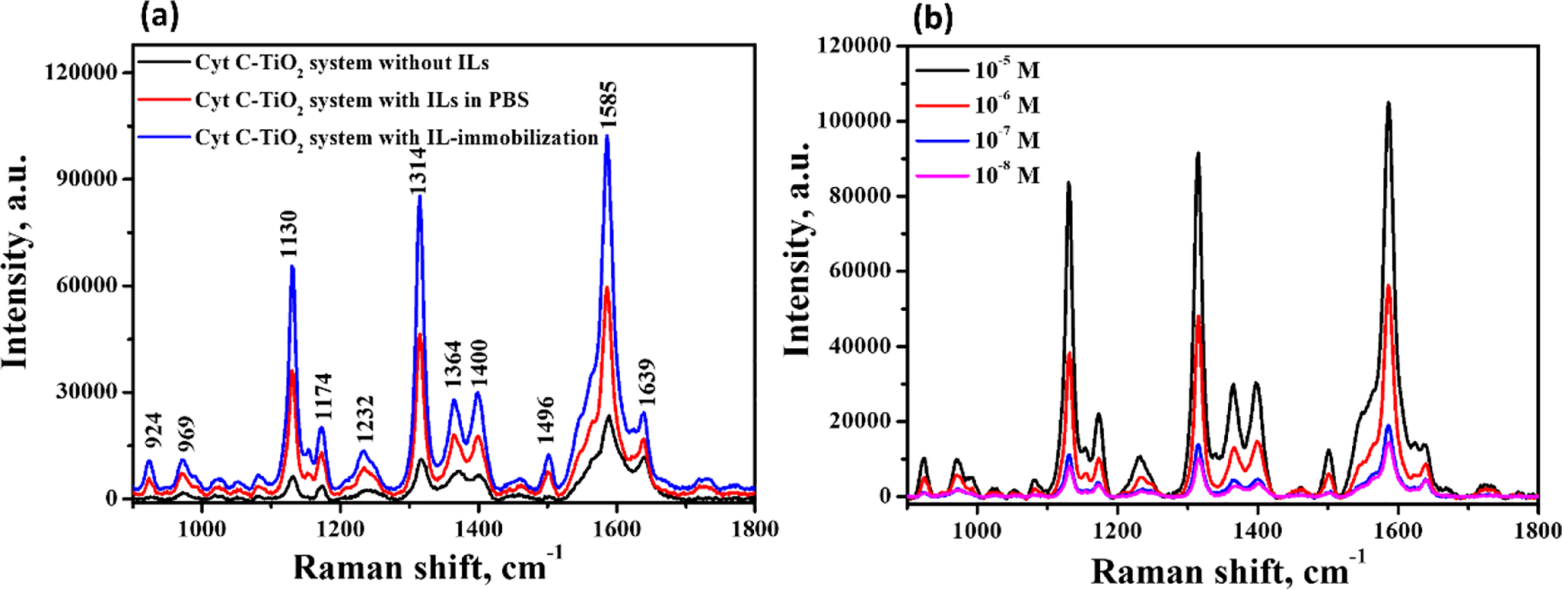
SERS spectra of (a) Cyt *c* (5 × 10^–4^ M) on TiO_2_ in three different systems (without ILs, with ILs in PBS, and with IL-immobilization); and (b) Cyt *c* with different concentrations on the TiO_2_.

In this work, the SERS measurements of Cyt *c* molecules on TiO_2_ nanotube arrays in three different systems were performed, as shown in Figure 3a. First, the SERS spectra of Cyt *c* molecules adsorbed on TiO_2_ nanotube array display the same vibrational band patterns as those for Cyt *c* molecules in the solution (Figure S2). The intensity of Cyt *c* in the solution was found to be extremely low when compared with that of Cyt *c* on the SERS substrate. According to the assignments and band locations for the SERS spectra of Cyt *c* on TiO_2_ nanotube array, the characteristic peaks in the spectrum of ν_10_(B_1g_) at 1639 cm^–1^, ν_3_(A_1g_) at 1496 cm^–1^, and ν_4_(A_1g_) at 1364 cm^–1^, correspond to the oxidized native states of Cyt *c*, indicating the biological activity of Cyt *c* molecules on TiO_2_ nanotube.^51^ Meanwhile, the seemingly negligible Raman signal of [Cho][Pro] on TiO_2_ nanotube array indicated that the effect of ILs on the Cyt *c* enhancement could be excluded (see Figure S3). Both the UV–vis adsorption spectra and photoluminescence (PL) spectra showed that the TiO_2_ nanotube array without and with IL-immobilization possessed almost the same light absorption behavior and the same separation efficiency in the electron and holes (see Figure S4), indicating no effects from the ILs on the optical properties of TiO_2_ substrate before the measurement. Second, we observed that the SERS performance of Cyt *c* on the TiO_2_ nanotube followed the order: with ILs immobilized on TiO_2_ system > with ILs in PBS system > without ILs system. The detailed normal mode assignments and band locations for the SERS spectra of Cyt *c* adsorbed on TiO_2_ nanotube array are listed in the SI (see Table S1). The most intensive peaks of Cyt *c* at the substrates for three different systems in the spectrum are the ν_19_(A_2g_) mode at 1585 cm^–1^, the ν_21_(A_2g_) mode at 1314 cm^–1^, and the ν_22_(A_2g_) mode at 1130 cm^–1^, respectively. The same band location indicated that the conformation and orientation of Cyt *c* on the TiO_2_ nanotube were consistent with each other for the studied three different systems.

According to the SERS performance, the introduction of ILs can realize the improvement of the SERS performance of Cyt *c*, especially with the ILs immobilized on the TiO_2_ substrate. Here, the TiO_2_ nanotube array with IL-immobilization was chosen to study the limit of detection, where the Cyt *c* content changed from 10^–5^ M to 10^–9^ M. As shown in Figure 3b, the Raman scattering enhancement can be detected even at 10^–9^ M, indicating that the detected sensitivity is far superior to that in most other related systems where the original or modified semiconductors were used.^19,52,53^ Therefore, through the introduction of ILs to improve the SERS performance of the semiconductors, excellent detection sensitivity can be observed at significantly low contents of protein. This clearly demonstrates that combining ILs with the TiO_2_ surface is beneficial and motivates its use in designing an interface or system for trace detection while lowering the limit of detection.

Meanwhile, the SERS performances of Cyt *c* on TiO_2_ were investigated for at least three batches to verify the stability and reproducibility of the ILs-immobilized TiO_2_ SERS substrate (see Figure S5). Furthermore, as EF is the already the widely accepted standard in the evaluation of SERS performance, its value was further determined according to the AFM-based molecular force in the following section to compare these three distinct SERS enhancements and to shed light on the mechanism at the molecular level.

### 3.3 Determination of the Enhancement Factor (EF)

According to the methods proposed,^54^ EF can be determined according to eq 1:
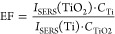
1where *C*_TiO2_ and *C*_Ti_ (mol·cm^–2^) are the adsorption capacity of Cyt *c* molecules on TiO_2_ surface and Ti substrate, and *I*_SERS(TiO2)_ and *I*_SERS(Ti)_ represent the band intensities of *ν*_21_ (A_2g_) at 1314 cm^–1^, respectively. However, for the proteins adsorbed on the small solid film surface (e.g., TiO_2_ nanotube), the number of adsorbed protein molecules (*C*_TiO2_) is usually extremely low and difficult to obtain directly. On the basis of our previous work, detection of the trace amounts of proteins adsorbed on the solid film surfaces can be obtained by the AFM-quantified adhesion force.^42^

For a molecular-level understanding of the mechanism, quantifying the molecular force of Cyt *c* on the TiO_2_ nanotube array in three different systems should be addressed first, where the values are obtained based on the combination of the adhesion force per unit contact area and the protein adsorption capacity per unit area, according to our previous work.^39^ As we discussed above, on the small film-like TiO_2_ nanotube array substrate, it is difficult to obtain the capacity of protein adsorption due to the trace amount. In this work, the mesoporous TiO_2_ microparticles with different surface areas were used to provide the molecular force, and the provided molecular force is independent of the geometric structure of the materials as revealed in our previous work.^42^ The results from the XRD measurements indicated that both the TiO_2_ nanotube arrays and the mesoporous TiO_2_ microparticles are the TiO_2_ with the anatase crystals but different nanostructures (see Figure S6). Therefore, the molecular force of Cyt *c* with TiO_2_ nanotube arrays is equal to that of Cyt *c* with mesoporous TiO_2_ microparticles.

#### 3.3.1 Quantification of the Molecular Force

##### 3.3.1.1 Quantification of Adsorption

**Figure 4:**
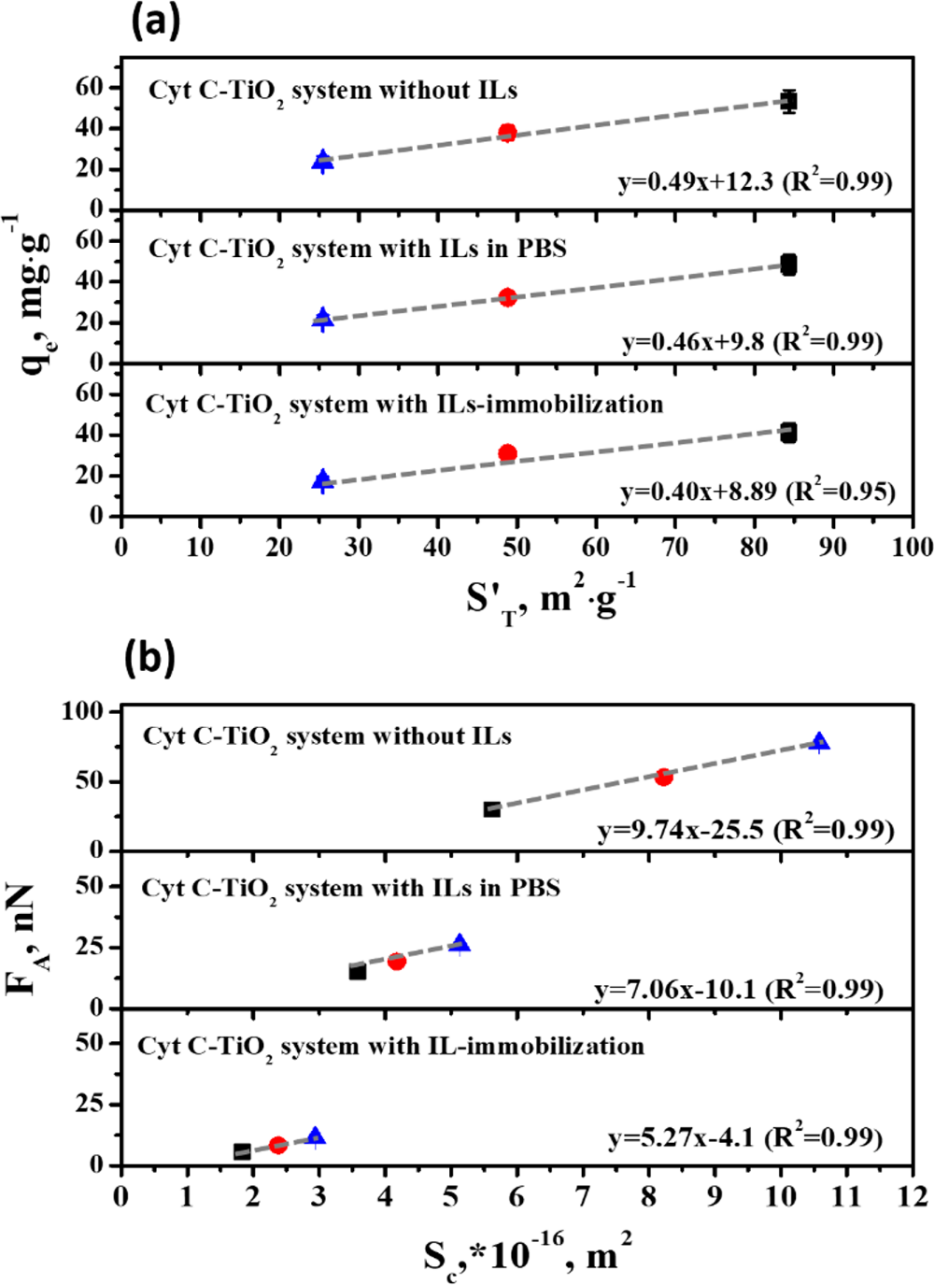
(a) The relationships between adsorption capacity of Cyt *c* on TiO_2_ and the effective surface area of TiO_2_ microparticles; (b) the relationships between adhesion force and effective contact area of Cyt *c* with TiO_2_ in three different systems. (from above: without ILs, with ILs in PBS, with IL-immobilization)

The mesoporous TiO_2_ microparticles with different effective surface areas (see Table S2) were chosen to study the adhesion force and the protein adsorption capacity. The adsorption capacities showed that the adsorption amounts of Cyt *c* on TiO_2_ microparticles with different effective surface areas were 63.3, 38.0, and 23.9 mg·g^–1^, respectively (see Table S3). Meanwhile, after the introduction of the ILs [Cho][Pro], the adsorption amounts of Cyt *c* on TiO_2_ microparticles with different effective surface areas were 54.4, 32.2, and 21.4 mg·g^–1^, corresponding to the system with ILs in PBS (see Table S3). Whereas, the adsorption amounts were down to 41.3, 30.8, and 17.4 mg·g^–1^, respectively, corresponding to the systems with ILs immobilized on TiO_2_ surface (see Table S3). To obtain the adsorption capacity of the Cyt *c* molecules on TiO_2_ per unit surface area, the effective surface areas of these mesoporous TiO_2_, i.e., the part that can be used for Cyt *c* adsorption effectively, was estimated based on the BET surface area of TiO_2_ provided by the pores larger than the size of the Cyt *c* molecule (>3.3 nm) (*S*_T_ → *S′*_T_, m^2^·g^–1^, Table S2). The amount of the Cyt *c* molecules adsorbed on each TiO_2_ per unit surface area (*q*_e_/*S′*_T_ in mg·m^–2^) was obtained based on the linear relationship between *S’*_T_ and *q*_e_ in Figure 4a. The corresponding slopes were 0.67, 0.57, and 0.40 for the systems without ILs, with ILs in PBS, and with ILs immobilized on TiO_2_, respectively. It was found that the adsorption capacity decreased after the introduction of [Cho][Pro], especially for the Cyt *c* adsorption on ILs-immobilized TiO_2_, which was found to be the lowest compared with the other systems. A deeper analysis was given after the quantification of the molecular force.

##### 3.3.1.2 AFM Adhesion Measurement and Quantification

The adhesion force of Cyt *c* with mesoporous TiO_2_ (*F*_A_ in nN) was measured with AFM, which represented the total interaction force between Cyt *c* clusters and the TiO_2_ surface, corresponding to the maximum force jump during retraction obtained from the force–distance curves. The adhesion forces of Cyt *c* were 11.4, 26.2, and 77.6 nN on TiO_2_ with different effective surface areas (see Table S4), respectively. However, the adhesion forces of Cyt *c* on TiO_2_ with different effective surface areas, which corresponded to the systems with ILs in PBS and with ILs-immobilization, were 8.3, 19.3, 53.2 nN, and 5.6, 15.3, 30.1 nN, respectively (see Table S4), indicating that the introduction of ILs weakened the interaction strength between Cyt *c* and TiO_2_ surface. The effective contact area (*S*_*c*_ in m^2^) between Cyt *c*-modified AFM tip and TiO_2_ surface was calculated based on the Hertz and Johnson–Kendall–Roberts (JKR) theories. Then the adhesion force of the Cyt *c* interacting with each TiO_2_ per unit contact area (*F*_A_*/S*_c_ in nN/m^2^) was obtained based on the linear relationship in Figure 4b, where the slopes were 9.74, 7.06, and 5.27 for the systems without ILs, with ILs in PBS, and with ILs-immobilized on TiO_2_, respectively.

##### 3.3.1.3 Molecular Force

**Figure 5:**
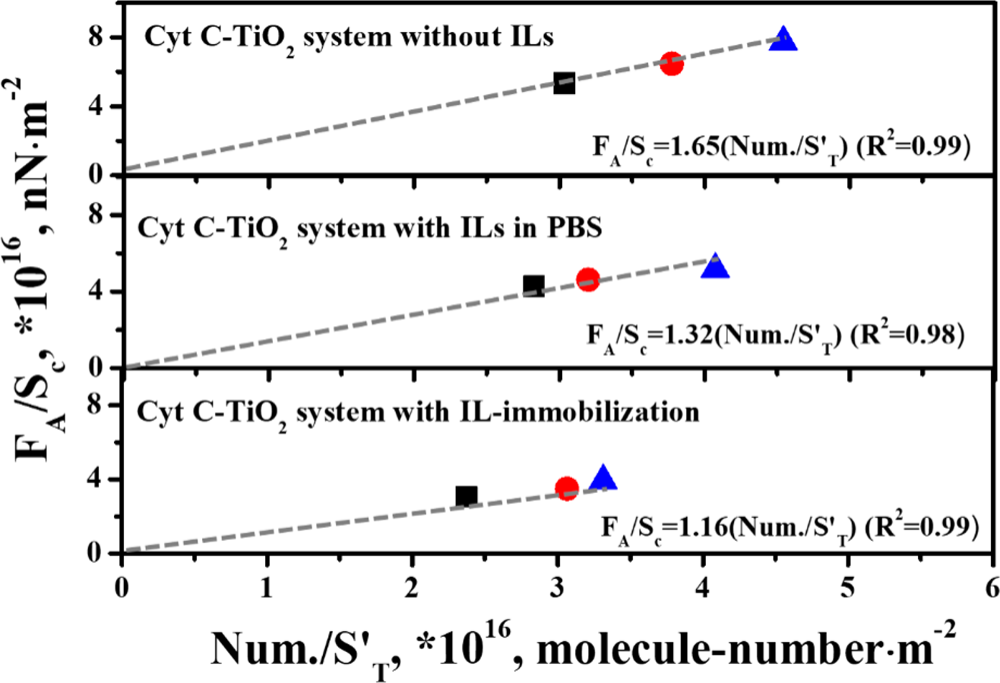
Relationship between adhesion force of Cyt *c* molecules on TiO_2_ per unit contact area and the number of Cyt *c* molecules adsorbed on TiO_2_ per unit area in three different systems (From above: without ILs, with ILs in PBS, with IL-immobilization).

As mentioned previously, the molecular force (*F*_s_) of one single Cyt *c* molecule interacting with TiO_2_ surface in three different studied systems could be obtained based on the combination of the adhesion force per unit contact area and the adsorption capacity per unit area. The relationships between *F*_A_/*S*_c_ (nN·m^–2^) and Num./*S′*_T_ (number-molecule·m^–2^) in three different systems were established to acquire the molecular force, as shown in Figure 5. The slope for each system represents the corresponding molecular force of one single Cyt *c* molecule interacting with the TiO_2_ surface, where the values were 1.65, 1.32, and 1.16 nN corresponding to the systems without ILs, with ILs in PBS, and with ILs immobilized on TiO_2_, respectively. The molecular force of Cyt *c* interacting with TiO_2_ decreased with the introduction of ILs, and the systems of ILs immobilized on TiO_2_ was found to be the lowest, indicating again that the existence of ILs weakened the Cyt *c*–TiO_2_ interaction strength. Whereas, the SERS measurements in Figure 3 showed that the improved performance of Cyt *c* on TiO_2_ with ILs-immobilization was the highest. As a result, the SERS performance was not always found to be proportional to the molecular force, which was further explained based on a hypothetical mechanism in the following section.

#### 3.3.2 Quantification of the EF Values

To quantify the EF values, the adhesion forces of Cyt *c* with TiO_2_ nanotube array (*F*_n_ in nN) in the three different systems were measured by AFM, whereby the values of 90.9, 27.3, and 11.9 nN were obtained. The effective contact area (*S′*_c_) between Cyt *c* molecules and TiO_2_ nanotube array was determined according to the Hertz and JKR theories (see Table 1). Then the adhesion force of Cyt *c* on TiO_2_ nanotube array per unit contact area was obtained with the values of 7.73 × 10^16^, 5.18 × 10^16^, and 3.93 × 10^16^ nN·m^–2^, respectively. Furthermore, the numbers of Cyt *c* molecules adsorbed on TiO_2_ nanotube arrays per unit area (*C*_TiO2_) were obtained based on the quantification of (*F*_n_/*S′*_c_)/(*F*_s_) with the values of 7.78 × 10^–12^, 6.52 × 10^–12^, and 5.62 × 10^–12^ mol·cm^–2^, corresponding to the three different systems (without ILs, with ILs in PBS, and with ILs-immobilization), respectively.

**Table 1:** Adhesion Force (*F*_n_), Effective Contact Area (*S′*_c_), and Numbers (*C*_TiO2_) of Cyt *c* on TiO_2_ Nanotube Array in Three Different Systems

sample	*F*_*n*_ (nN)	*S′*_*c*_ (m^2^)	*C*_TiO2,_ (mol·cm^–2^)
Cyt *c*-TiO_2_	90.9 ± 3.7	1.18 × 10^–15^	7.78 × 10^–12^
Cyt *c*-ILs/TiO_2_	27.3 ± 3.2	5.27 × 10^–16^	6.52 × 10^–12^
Cyt *c*/ILs-TiO_2_	11.9 ± 2.9	3.03 × 10^–16^	5.62 × 10^–12^

Considering *I*_SERS(Ti)_ and *C*_Ti_ are the same for Cyt *c* on Ti substrate, the values of *I*_SERS(TiO2)_*/I*_SERS(Ti)_ and *C*_TiO2_/*C*_Ti_ are listed in Table 2. The quantified values of EF for Cyt *c* on TiO_2_ nanotube array without ILs, with ILs in PBS, and with IL-immobilization are 2.30 × 10^4^, 6.17 × 10^4^, and 1.19 × 10^5^, respectively. It was shown that the introduction of ILs improved the detection performance and the ILs immobilized TiO_2_ system provided an excellent detectable surface enhancement. The EF values of the detected molecules on those semiconductor materials (i.e., TiO_2_) are generally in the order of 10^2^–10^3^, while, in this work, the introduction of ILs enhanced the SERS performance and reached the order of 10^5^. The mechanism was discussed in the following section.

**Table 2:** Parameters and the Calculated EF of Cyt c on the TiO_2_ Nanotube Arrays in Three Different Systems

sample	*I*_SERS(TiO2)_/*I*_SERS(Ti)_	*C*_Ti_/*C*_TiO2_	EF
Cyt *c*-TiO_2_	6.58 × 10^4^	0.35	2.30 × 10^4^
Cyt *c*-ILs/TiO_2_	1.47 × 10^5^	0.42	6.17 × 10^4^
Cyt *c*/ILs-TiO_2_	2.47 × 10^5^	0.48	1.19 × 10^5^

### 3.4 Mechanism

#### 3.4.1 Effect of ILs on Molecular Force and SERS Performance

**Figure 6:**
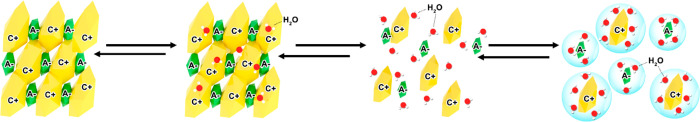
Scheme of the dissociation and hydration of cation and anion of [Cho][Pro] in water solution.

**Figure 7:**
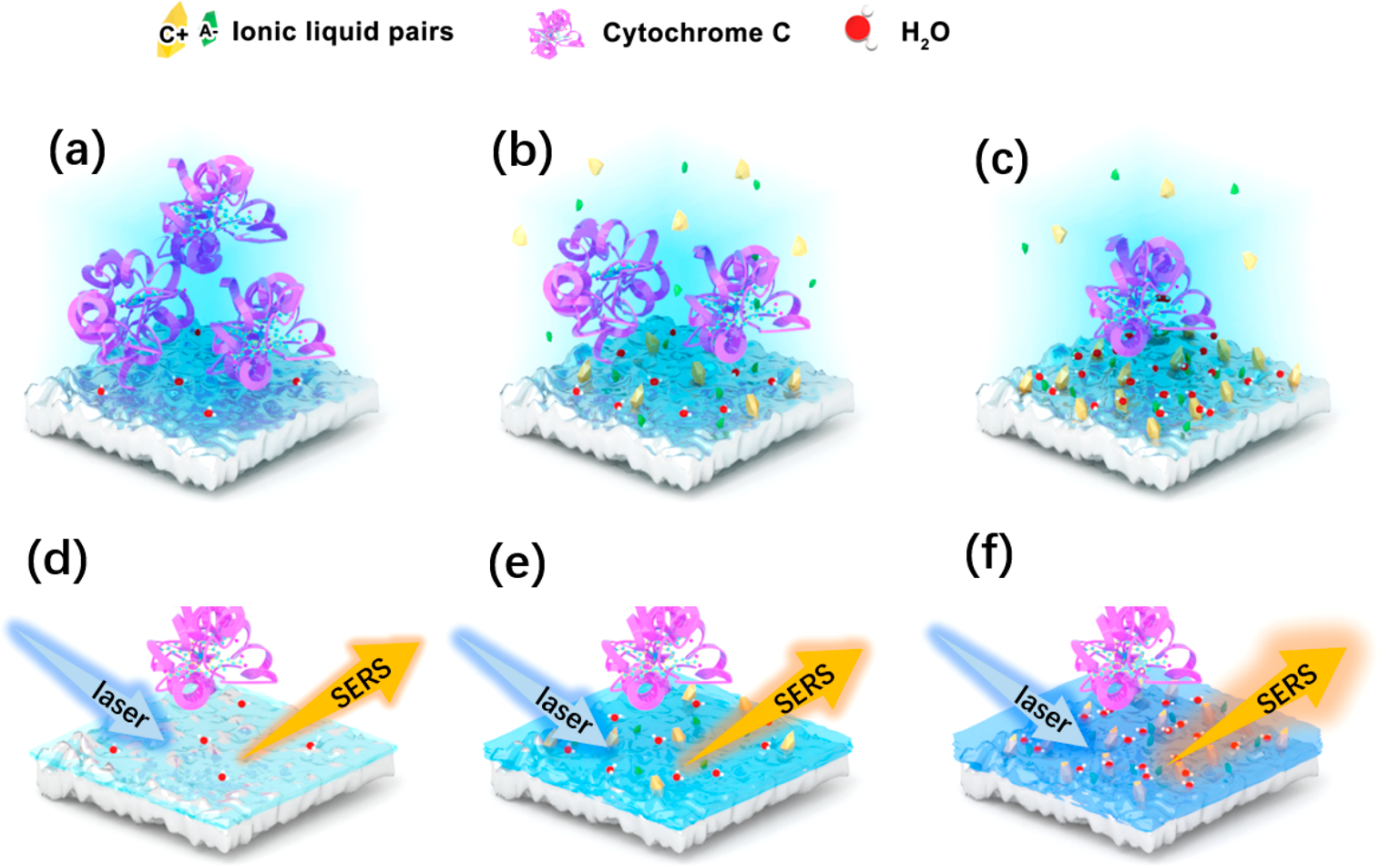
Scheme of the different interaction behavior of Cyt *c* with TiO_2_ in the system (a) without ILs, (b) with ILs in PBS, and (c) with ILs immobilized on TiO_2_ surface; and the scheme of effects of hydration of ILs on SERS enhancement between Cyt *c* and TiO_2_ surface in the system (d) without ILs, (e) with ILs in PBS, and (f) with ILs immobilized on TiO_2_ surface.

When the ILs ([Cho][Pro]) were added into the Cyt *c* solution with a large amount of water as the solvent, the ILs could be dissociated into cations and anions. A complete or partial dissociation and hydration of [Cho][Pro] took place, and thus the mixture could be treated as classical electrolyte solutions,^55,56^ as shown in Figure 6. Furthermore, the counterion release model and the hydration mechanism were applied to analyze the interaction of the protein with TiO_2_ in these three systems (see Figure 7a–c). On the one hand, when the negatively charged TiO_2_ is immersed into a solution containing dissociated negative and positive ions, the vicinity of the surface will be changed by these ions. The ions with the same sign as the surface charges will be repelled into the bulk, and those with the opposite sign as the surface charges will be drawn toward the surface, leading to the formation of a shielding layer covered with cations formed from the electrolyte solution and ILs and the decrease of the adsorption of positively charged Cyt *c* molecules on TiO_2_ surface. On the other hand, the hydration of ions is the key to protein adsorption. Simulations show that the cation (choline) is hydrated by H_2_O, and aggregates much closer to the negatively charged TiO_2_ surface (see Figure S7), implying that a hydration layer induced by the cation of ILs is formed.^57^ Therefore, with introducing ILs in PBS, the molecular force of Cyt *c* on TiO_2_ was decreased due to the existence of the hydrated cation adsorbed on TiO_2_ surface.

To further investigate, the UV–vis adsorption spectra and PL measurements were determined. It showed that the system of Cyt *c* adsorbed on TiO_2_ with ILs provided a better light absorption than the one without ILs (see Figure S8a). Meanwhile, according to the PL spectra, the signal intensity of Cyt *c* on the TiO_2_ with IL-immobilization was weakened compared with the one without ILs (see Figure S8b). It indicated that the introduction of ILs into TiO_2_-based SERS systems provided a higher separation efficiency in the holes and electron, evidencing a better property in the electron transport. Therefore, the dissociation of ILs into cations and anions and the formation of the hydration layer increased the ionic conductivity and electro-transfer properties, leading to the enhanced SERS performance (Figure 7d–f).

For the Cyt *c* adsorption in the system with ILs immobilized on TiO_2_, a strong hydration layer could also be formed on TiO_2_ surface after immersion into PBS. Also, the hydration strength of the ILs immobilized on the TiO_2_ surface was stronger than that for the ILs adsorbed to the TiO_2_ surface from the solution, due to the weaker diffusion of the hydrated ions for the ILs immobilized on TiO_2_ compared with the ILs in the solution (Figure 7c).

In summary, the molecular interaction of Cyt *c* on IL-immobilized TiO_2_ was found to be weaker than that on TiO_2_ with ILs in PBS, while the dissociation and hydration of ILs effectively increased the electron transfer. The increased electron transfer ability is most likely the main reason to improve the SERS intensity. To further confirm this, the decreased molecular interaction was further verified with the friction measurements.

#### 3.4.2 Verification of Molecular Interactions by Friction Measurements

**Figure 8:**
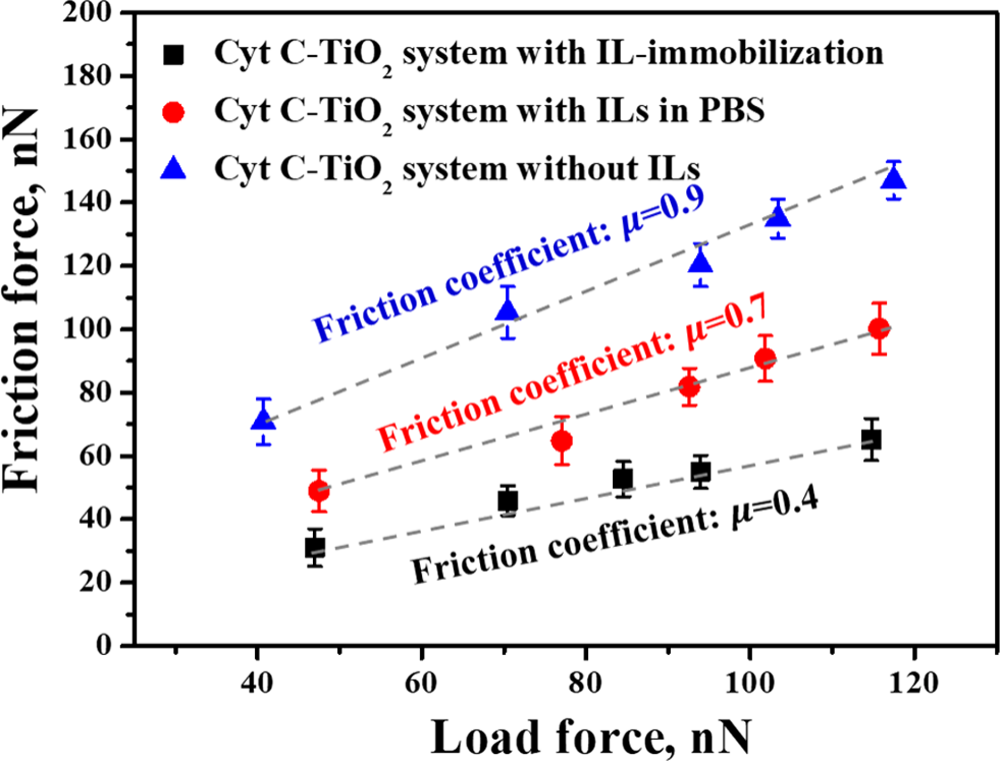
Friction force vs load force of Cyt *c* with TiO_2_ in three different systems.

To further verify the effects of hydrated ILs on the protein interaction with TiO_2_ nanotube array, the friction force was studied. The friction force vs load force between the Cyt *c*-tip and TiO_2_ nanotube array in three different systems was measured by AFM, as shown in Figure 8. The friction coefficient was quantified by dividing the friction force by the load force. The results showed that the friction coefficient of Cyt *c* with TiO_2_ nanotube array decreased with the introduction of ILs. This trend is found to be consistent with the discussions of the molecular force. The friction force can be strengthened due to the larger molecular force requiring more energy to break the force to separate the protein-tip from the substrate. The value of the friction coefficient was about 0.9 for the system without ILs, whereas the values for the system with ILs in PBS and with ILs immobilized on TiO_2_ nanotube array were 0.7 and 0.4, respectively. However, the decreased friction coefficient was found in the system with ILs, where the stronger hydration and dissociation of the ILs occurred. This is also consistent with the literature that the hydration of the dissociated ions could improve the lubrication, leading to the decrease of the friction coefficient.^58^

## 4 Conclusions

In this work, hydrophilic ILs [Cho][Pro] were added into PBS and immobilized on TiO_2_ surface, respectively, to study the SERS performance of Cyt *c* on TiO_2_ surface and compared with the system without ILs. A very low limit of detection down to 10^–9^ M was achieved after the introduction of ILs. The obtained values of EF were 2.30 × 10^4^, 6.17 × 10^4^, and 1.19 × 10^5^, corresponding to the system without ILs, with ILs in PBS, and with ILs immobilized on TiO_2_, respectively, showing that the introduction of ILs into the system did clearly enhance the SERS performance. To understand the mechanism on the molecular level, the molecular forces of Cyt *c* with TiO_2_ surface in three different systems were determined by measuring the adhesion force and adsorption capacity. Due to the hydration of both the cations and anions of the ILs, the molecular forces were weakened after the introduction of ILs, but did improve the electron transfer ability of ILs instead, leading to an excellent SERS performance. Meanwhile, a weaker diffusion of the hydrated IL ions immobilized on TiO_2_ results in a greater EF value compared with that caused by the ILs in the solution. These important hydration properties were also verified in the measured friction behavior. On the basis of our findings, the introduction of ILs did demonstrate the importance of adding an extra environment leading to a truly remarkable enhancement in the SERS performance in trace detection. Furthermore, the distinct SERS performance of other proteins on TiO_2_ with the introduction of ILs will be studied in our future work, especially meeting the demands on the trace detection of proteins in a variety of applications. The proposed method is also expected to stimulate further developments of using ILs in the SERS and related applications in bioanalysis, medical diagnosis, and environmental science to mention a few.

## Supporting Information Available

SEM and AFM images of TiO_2_ nanotube; Raman spectra of Cyt *c* in solution; Raman signal of [Cho][Pro]; UV–vis absorption spectra and PL measurements of substrates; three batches of SERS measurements of Cyt *c* on TiO_2_; XRD patterns; number density profile of ions on TiO_2_ surface; resonance Raman scattering, the normal mode assignments, and band locations; BET results; Table S3, results of adsorption capacity; results of adhesion force; and UV–vis absorption spectra and PL measurements of Cyt *c* on TiO_2_ (PDF)
