# Supplementary material for: Hydrated Ionic Liquids Boost the Trace Detection Capacity of Proteins on TiO2 Support
Source: Langmuir. 2021 Apr 16;37(16):5012–21. doi: 10.1021/acs.langmuir.1c00525 (PMC8154861; doi:10.1021/acs.langmuir.1c00525)
Supplement: Supplementary file 1 — la1c00525_si_001.pdf [file la1c00525_si_001.pdf]

Supporting information

Hydrated ionic liquids boost the trace detection capacity of proteins on TiO_2 support

Yihui Dong[a]*, Aatto Laaksonen[b,c,d,e], Feng Huo[a], Qingwei Gao[f], and Xiaoyan Ji[b]*

[a]*Beijing Key Laboratory of Ionic Liquids Clean Process, CAS Key Laboratory of Green Process and Engineering, State Key Laboratory of Multiphase Complex Systems, Institute of Process Engineering, Chinese Academy of Sciences, Beijing 100190, P. R. China.*

[b]*Energy Engineering, Division of Energy Science, Luleå University of Technology, 97187 Luleå, Sweden.*

[c]*Department of Materials and Environmental Chemistry, Arrhenius Laboratory, Stockholm University, Stockholm SE-10691, Sweden.*

[d]*State Key Laboratory of Materials-Oriented Chemical Engineering, Nanjing Tech University, Nanjing 210009, P. R. China.*

[e]*Centre of Advanced Research in Bionanoconjugates and Biopolymers, Petru Poni Institute of Macromolecular Chemistry, Iasi 700487, Romania.*

[f]*State Key Laboratory of Chemical Engineering and School of Chemical Engineering, East China University of Science and Technology, Shanghai 200237, P. R. China.*

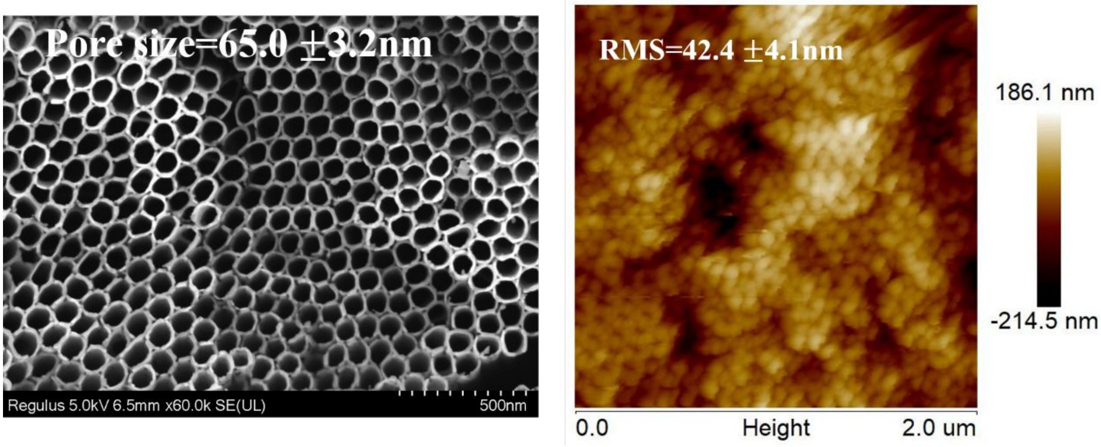

Figure S1. SEM results and AFM topography of TiO_2 nanotube array.

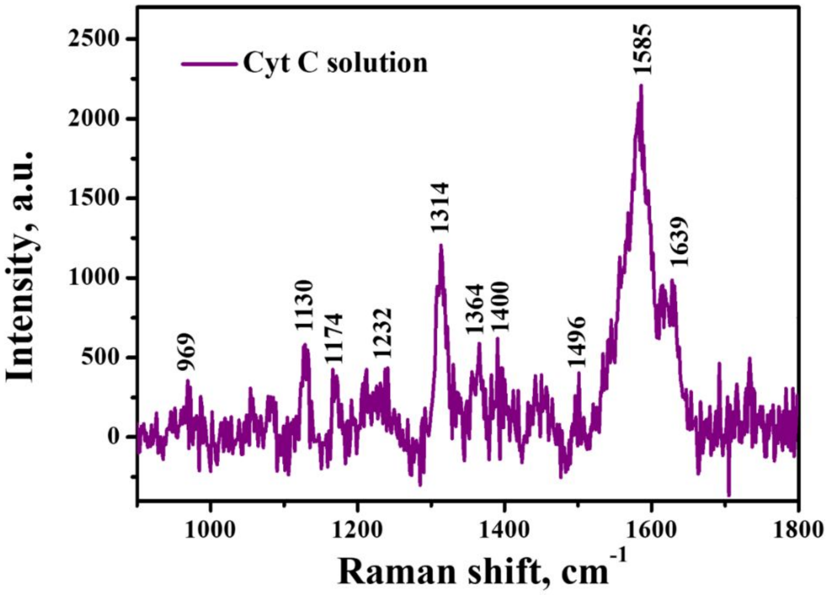

Figure S2. Raman spectra of Cyt C in solution (5×10^{-4} M).

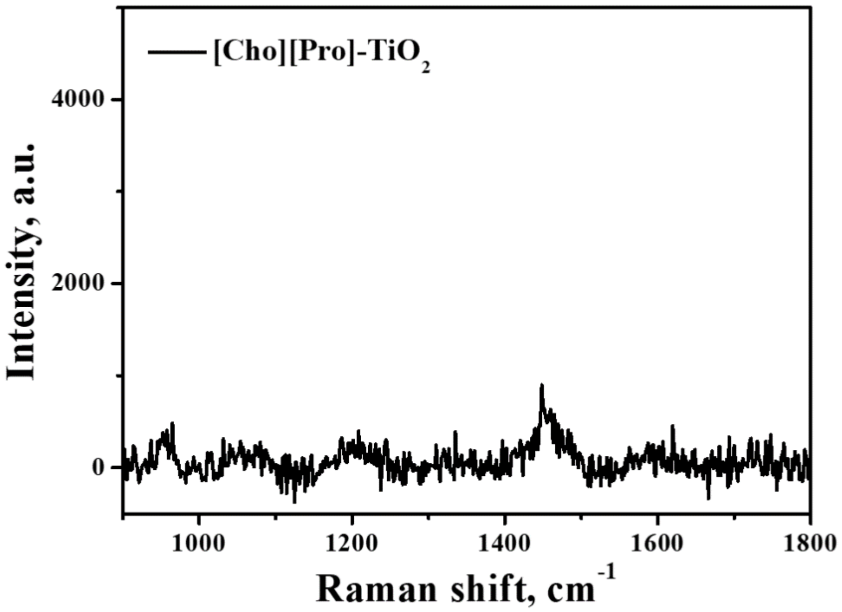

Figure S3. Raman signal of [Cho][Pro] on TiO_2 nanotube arrays.

The Ultraviolet-visible (UV-vis) adsorption spectra were recorded on a Shimadzu UV-3600 UV-vis spectrometer. The barium sulfate ($BaSO_4$) was used as an internal standard. The measurement of TNAs was over a wavelength range of 300-800 nm. The Photoluminescence (PL) spectra of samples were investigated and recorded by PL tool operating with 310 nm laser (helium–cadmium laser) in association with a spectrophotometer (Edinburgh FLS 980).

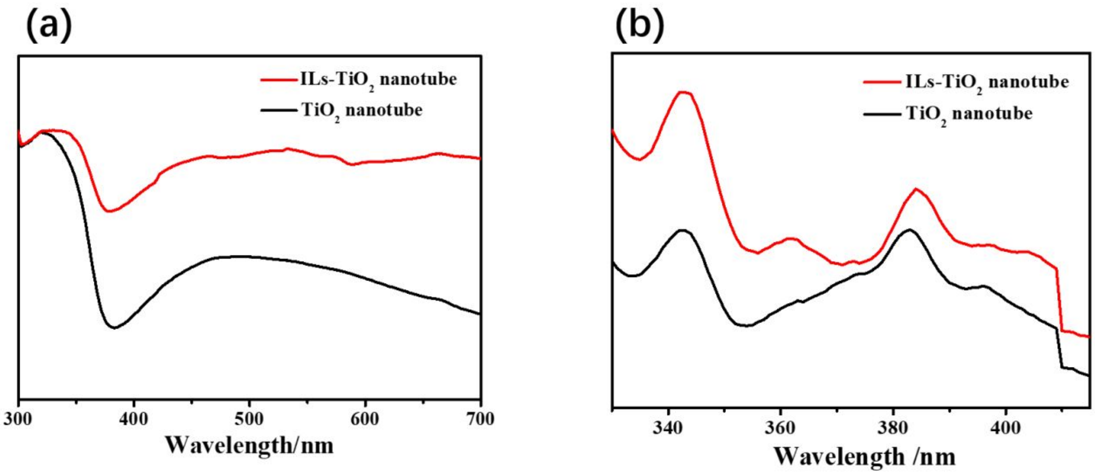

Figure S4. (a) UV−vis absorption spectra and (b) Photoluminescence (PL) study of TiO_2 nanotube and ILs-immobilized TiO_2 nanotube.

Table S1. Resonance Raman scattering (RRS), band locations, and the normal mode assignments for SERS spectra of Cyt C.

RRS	Mode	Symmetry	Local coordinate
924	ν_{46}	E_u	δ(pyr deform)$_{asym}$
969	/	A_{2u}	$\upsilon(C_c\text{-}C_d)_{6,7}$
1130	ν_{22}	A_{2g}	υ(pry half-ring)$_{asym}$
1174	ν_{30}	B_{2g}	υ(pry half-ring)$_{sym}$
1232	ν_{13}	B_{1g}	$\delta(C_m\text{-}H)$
1314	ν_{21}	A_{2g}	$\delta(C_m\text{-}H)$
1364	ν_4	A_{1g}	υ(pry half-ring)$_{sym}$
1400	ν_{20}	A_{2g}	υ(pry quarter-ring)
1496	ν_3	A_{1g}	$\upsilon(C_a\text{-}C_m)_{sym}$
1585	ν_{19}	A_{2g}	$\upsilon(C_a\text{-}C_m)_{asym}$
1639	ν_{10}	B_{1g}	$\upsilon(C_a\text{-}C_m)_{asym}$

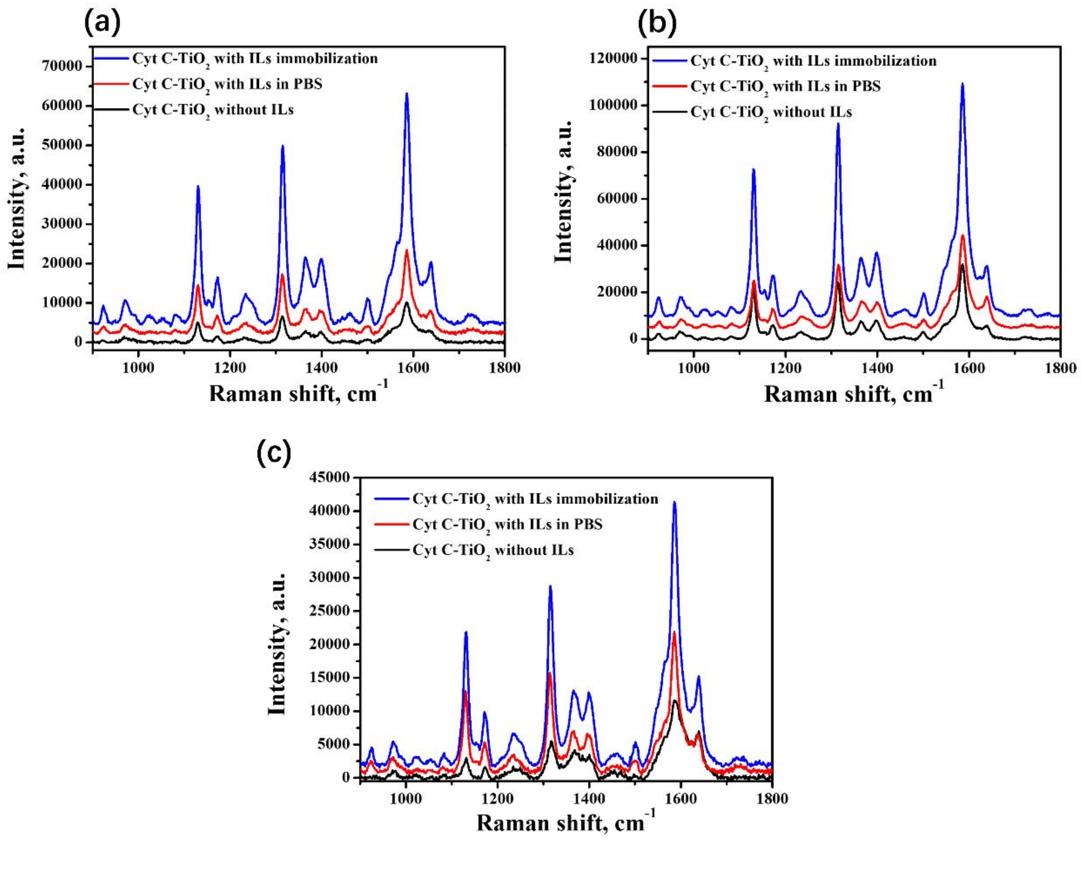

Figure S5. Three batches of the repetitive SERS measurements of Cyt C molecules on TiO_2 under three different systems.

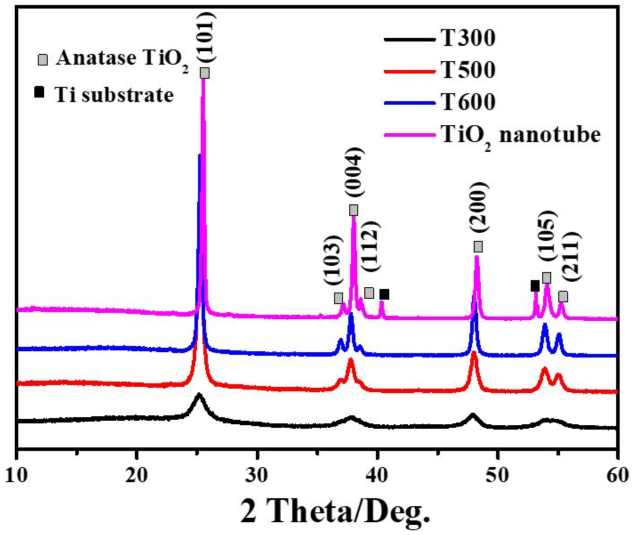

Figure S6. XRD patterns of mesoporous TiO_2 and TiO_2 nanotube array.

Table S2. BET pore size and surface area (S_T) and effective surface area (S'_T) of TiO_2 deducted by the part related to the size of Cyt C.

Sample	Pore size, nm	S_T, $m^2 \cdot g^{-1}$	S'_T, $m^2 \cdot g^{-1}$
T300	8.35	140.6	84.36
T500	19.7	50.53	48.81
T600	32.5	25.77	25.46

The adsorption capacity of Cyt C on TiO_2 was achieved by measuring the adsorption of Cyt C from the aqueous solution (2 $mg \cdot mL^{-1}$, a 0.01 M PBS at pH =7.2). Three different systems were considered: (1) TiO_2 samples were added to a centrifuge tube containing 20 mL Cyt C solution; (2) TiO_2 samples were added to a centrifuge tube containing 20 mL Cyt C solution and 0.01g IL; (3) ILs-immobilized TiO_2 samples were added to a centrifuge tube containing 20 mL Cyt C solution. During the adsorption process, the samples in the centrifuge tubes were kept in a water bath at 30 °C and shaken at 180 rpm for 72 h. Then, Cyt C adsorbed on the substrate samples were separated by spinning the mixtures in sealed centrifuge tubes at 8000 rpm for 10 min. The supernatant concentration of Cyt C was determined by measuring the Cyt C

absorbance with a UV-vis spectrophotometer at λ=409 nm. The amount of Cyt C bound to the substate sample was calculated from the difference in the initial and final protein concentrations.

Table S3. Adsorption capacity ($mg \cdot g^{-1}$) of Cyt C on mesoporous TiO_2 microparticles under three different systems.

Sample	With ILs-free	With ILs in solution	With ILs-immobilization
T300	63.3	54.4	41.3
T500	38.0	32.2	30.8
T600	23.9	21.4	17.4

Table S4. Adhesion force (nN) of Cyt C on mesoporous TiO_2 microparticles under three different systems.

Sample	With ILs-free	With ILs in PBS	With ILs-immobilization
T300	11.4	8.3	5.6
T500	26.2	19.3	15.3
T600	77.6	53.2	30.1

Classical Molecular Dynamics simulations were performed using the GROMACS package. The force field and simulation protocols were found in the recent work [Journal of Molecular Liquids 319 (2020) 114298]. Three-dimensional periodic boundary conditions were applied, and the simulation box size of the initial configuration is 25.1 nm × 25.1 nm × 25.0 nm.

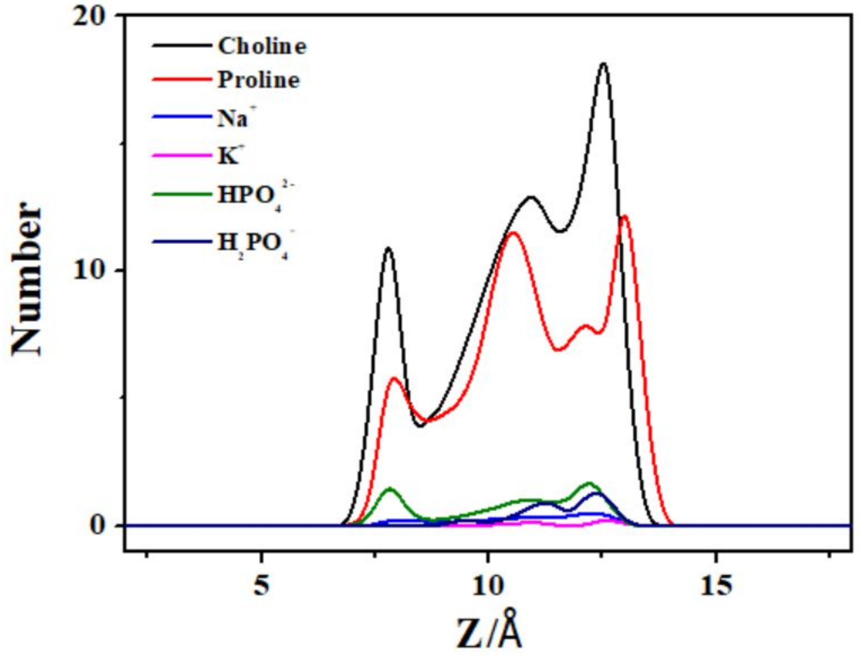

Figure S7. Number density profile of ions on TiO_2 surface.

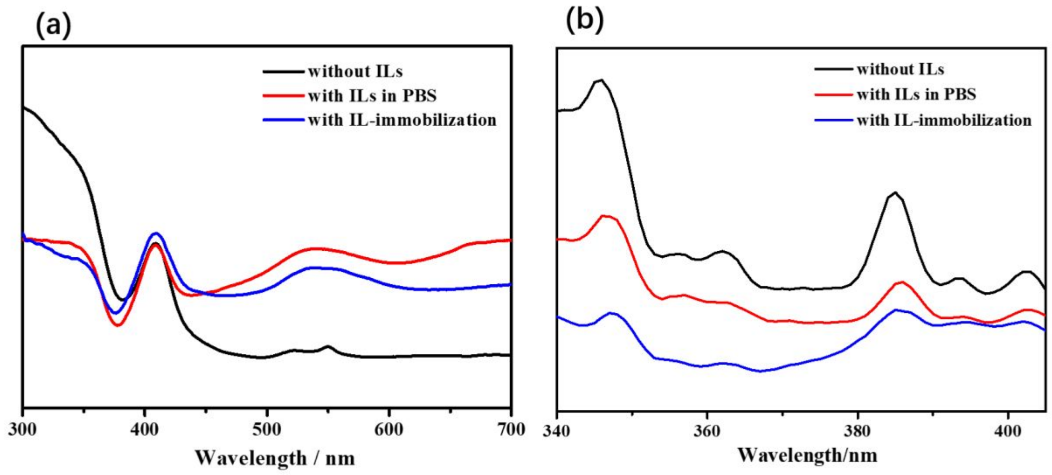

Figure S8. (a) UV−vis absorption spectra and (b) Photoluminescence (PL) study of Cyt C interacting with TiO_2 nanotube under three different systems.
